# Topical application of recombinant calreticulin peptide, vasostatin 48, alleviates laser-induced choroidal neovascularization in rats

**Published:** 2010-04-28

**Authors:** Youn-Shen Bee, Shwu-Jiuan Sheu, Yi-Ling Ma, Hsiu-Chen Lin, Wen-Tsang Weng, Hsiao-Mei Kuo, Huei-Chun Hsu, Chia-Hua Tang, Jau-Cheng Liou, Ming-Hong Tai

**Affiliations:** 1 Department of Ophthalmology, Kaohsiung Veterans General Hospital, Kaohsiung, Taiwan; 2 School of Medicine, National Yang-Ming University, Taipei, Taiwan; 3 Department of Biological Sciences, National Sun Yat-Sen University, Kaohsiung, Taiwan; 4 Department of Medical Education and Research, Kaohsiung Veterans General Hospital, Kaohsiung, Taiwan; 5 Department of Biomedical Sciences, National Sun Yat-Sen University, Kaohsiung, Taiwan

## Abstract

**Purpose:**

Vasostatin 48 (VS48) is a peptide of 48 amino acids derived from calreticulin. This study aimed to investigate the effects of topical application of VS48 eyedrops on experimental choroidal neovascularization (CNV).

**Methods:**

Recombinant VS48 was expressed and purified as a thioredoxin (TRX)-fused protein, TRX-VS48. The anti-angiogenic effects of TRX-VS48 were validated by migration and tube formation assays performed on cultured endothelial cells, and by rat aorta ring assays. CNV lesions were created in Brown Norway rats by laser-induced photocoagulation at day 1. After topical TRX-VS48 application for 21 days, the CNV lesions were monitored via either choroidal flat mounts on day 21 or by fluorescent angiography on days 21, 28, 35, and 42. CNV lesions were evaluated by histological analysis. The retinal function of animals was examined by electroretinogram (ERG) to evaluate the safety and therapeutic efficacy of TRX-VS48.

**Results:**

Application of TRX-VS48 inhibited the migration and tube formation of endothelial cells. TRX-VS48 inhibited the growth of sprouting vessels in aorta rings. ERG analysis revealed that topical TRX-VS48 application for 21 days had no effect on rat retinal functions. After CNV induction, topical TRX-VS48 application for 21 days significantly reduced the size of CNV, as assayed by flat mounts. Fluorescent angiography revealed that the CNV areas in TRX-VS48-treated eyes were significantly reduced compared with TRX-treated eyes on days 21, 28, 35, and 42. Histological analysis also revealed attenuated CNV lesions in TRX-VS48-treated eyes. Topical TRX-VS48 treatment significantly reversed the CNV-induced alterations in ERG parameters on day 35.

**Conclusions:**

Topical TRX-VS48 application suppressed laser-induced CNV in rats, thereby constituting a possible modality for ocular diseases due to excessive angiogenesis.

## Introduction

Age-related macular degeneration is the leading cause for visual impairment and blindness among the elderly in developed countries. The primary underlying cause for significant visual loss is choroidal neovascularization (CNV) [1]. Antiangiogenic treatment is a new trend for ocular neovascularization [2]. Several forms of antiangiogenic agents have been reported to be effective in inhibiting choroidal neovascularization in animal models [2-8]. This research has lead to the development of a vascular endothelial growth factor (VEGF)-neutralizing oligonucleotide aptamer (pegaptanib) [9], humanized anti-VEGF monoclonal antibody bevacizumab (Avastin) [10,11], and humanized anti-VEGF monoclonal antibody fragment (Ranibizumab) [12,13] for CNV treatment. In our previous studies, we demonstrated that gene delivery of angiogenesis inhibitor vasostatin (VS) attenuated CNV in a rat model [14,15]. Repeated long-term intravitreal delivery, as would be required for these antiangiogenic agents, runs the risk of significant local complications such as retinal detachment, endophthalmitis, vitreous hemorrhage, and cataract formation [16].

Vasostatin, the N-terminal domain (amino acids 1–180) of calreticulin, is a potent angiogenesis inhibitor isolated from culture supernatants of an Epstein-Barr virus-immortalized cell line. Recombinant VS was shown to inhibit basic fibroblast growth factor (bFGF) and VEGF-induced endothelial cell proliferation in vitro [17]. In our previous studies, intramuscular VS gene delivery led to elevated VS levels in the blood and inhibited tumor growth and CNV in animals [14,18]. Moreover, topical VS application potently abolished bFGF-induced cornea angiogenesis and inhibited laser-induced CNV in rat eyes [15,19]. Recently, we dissected the functional domain of VS into a peptide fragment of 48 residues, vasostatin 48 (VS48), which consists of residues 133–180 of calreticulin. In the present study, we validated the anti-angiogenic function of recombinant VS48, and evaluated the therapeutic potential of topical thioredoxin-fused vasostatin 48 (TRX-VS48) applications for treatment of laser-induced CNV in rat eyes.

## Methods

### Cloning, expression, and purification of recombinant vasostatin 48

The cDNA-encoding VS48 residues 133–180 of calreticulin were amplified from human vasostatin cDNA by PCR and subcloned into the NdeI and XhoI sites of the pET32 vector (Novagen Inc., Madison, WI) to yield the pET32-VS48 plasmid. For expression and purification, the pET32-VS plasmid was transformed into BL-21 cells (pLysS; Novagen Inc.) and the transformed cells were grown at 37 °C until log phase (optical density [OD]_600 nm_ of 0.5–0.9). Subsequently, cells were supplemented to induce protein expression and further cultured. The cell pellet was harvested by centrifugation at 6,000× g for 10 min at 4 °C, resuspended in binding buffer containing 20 mM phosphate buffer at pH 7.4, 20 mM imidazole, 150 mM NaCl, 1 mM EDTA, 1 mM PMSF, 1 µg/ml aprotinin, 1 µg/ml leupeptin, and 1 µg/ml pepstatin, and then homogenized by sonication. After centrifugation, the supernatant was mixed with Ni-NTA agarose (Qiagen GmbH, Germany). After washing the beads, the recombinant protein was eluted with buffer containing 20 mM phosphate buffer pH 7.4, 250 mM imidazole, and 150 mM NaCl, and it was then desalted by passing through a G-25 Sephadex column (Amersham Pharmacia Biotech, UK). The recombinant protein was passed through Detoxi-G gel (Pierce, Rockford, IL) so that the purified VS48 was free of endotoxin as analyzed by a *Limulus* amebocyte lysate assay (Sigma, St. Louis, MO).

### Western blot

The protein extract was isolated using radioimmunoprecipitation assay (RIPA) buffer (150 mM NaCl, 50 mM 4-(2-hydroxyethyl)-1-piperazineethanesulfonic acid (HEPES) pH 7.5, 1% Triton X-100, 10% glycerol, 1.5 mM MgCl_2_, 1 mM EGTA) containing protease inhibitor (Roche Applied Science, Indianapolis, IN). After separation via 15% sodium dodecyl sulfate PAGE (SDS–PAGE), the protein samples were transferred onto a polyvinylidene fluoride (PVDF) membrane using a blotting apparatus. The membrane was blocked with 5% milk in Tris-Buffered Saline Tween-20 (TBST) for 1 h then incubated with Hexa histidine-tag (His-Tag) antibodies (1:200 dilutions; Santa Cruz Inc., Santa Cruz, CA) for 1 h at room temperature. After incubation with a secondary antibody conjugated with horseradish peroxidase (HRP; 1:10,000 dilution in 5% milk) for 30 min, the signals on the membrane were detected using electrochemiluminescence (ECL)-plus luminol solution (Pharmacia, Piscataway, NJ) and exposed to X-ray film for visualization.

### Cell cultures

ECV304 endothelial cells were cultured in RPMI 1640 medium (Life Technologies, Rockville, MD) containing 100 μg/ml penicillin (Hyclone, Logan, UT), 100 μg/ml streptomycin (Hyclone), 10% fetal bovine serum (GIBCO BRL, Rockville, MD), and 2 mmol/l glutamine (Hyclone) under humidified conditions in 95% air and 5% CO_2_ at 37 °C.

### Tube formation assay

ECV 304 cells were seeded on matrigel-coated 24 well plates (BD Biosciences, San Jose, CA) at a 60% cell density. The cells were treated with TRX and TRX-VS48 protein at different concentrations (1, 5, and 10 µg/ml) and incubated for 5 h. All incubations were performed in an incubator at 37 °C and in 5% CO_2_ and 95% air. The number of tubes was counted under a bright-field phase contrast microscope. Only the complete ring structures created by 3–5 endothelial cells were counted as tubes.

### Migration assay

Trypsinized ECV 304 cells were used for migration assays using a Boyden's chamber, which is a two-chamber system. The upper and lower chambers were separated by a collagen-coated, 8-µm pore-sized polycarbonate membrane. ECV 304 cells were loaded in the upper well at 2×10^4^ cells per well with the TRX or TRX-VS48 protein. The lower well was filled with RPMI. The chambers were then incubated at 37 °C in 5% CO_2_ for 6 h. Cells migrated across the membrane and stuck to the lower part of the membrane. After the incubation, the polycarbonate membrane was fixed and stained with Giemsa stain (Sigma-Aldrich, Munich, Germany). Endothelial cell migration activity was quantified as the number of cells that migrated to the lower surface of the membrane.

### Rat aorta ring assay

This ex vivo angiogenesis assay was performed as described previously [20]. Briefly, the thoracic aortas were excised from 3-week-old Sprague-Dawley male rats and immediately placed into cold Dulbecco’s modified Eagle’s medium containing 10% FBS. Clotted blood inside the aorta was flushed with media, and the periadventitial fibroadipose tissue was removed. Aortas were then cut into cross-sectional rings about 1–1.5 mm in length. First, rings were placed on matrigel-plated 24-well plates and MCDB131 medium supplemented with antibiotic and incubated at 37 °C until the matrigel polymerized. The wells were then overlaid with 0.5 ml 10% serum of MCDB131 media containing 5 μg/ml TRX-VS48 or TRX, and the rings were maintained at 37 °C for up to 5 day. Vascular sprouting from each ring was examined daily on an Olympus microscope (20× magnification; Olympus, Tokyo, Japan), and digital images were obtained. The greatest distance from the aortic ring body to the end of the vascular sprouts (sprout length) was measured using NIH Image software (Scion Corp., Frederick, MD) at three distinct points per ring for three different rings per treatment group.

### Animals

Brown Norway pigmented rats and Sprague-Dawley male rats (200–250 g; National Animal Center, Taipei, Taiwan) were used. All rats were handled in accordance with the ARVO Statement for the Use of Animals in Ophthalmic and Vision Research. The experimental procedures and animal care and uses were approved by the Institutional Animal Care and Use Committee (IACUC). The rats were anesthetized by intramuscular injection of an equal-volume mixture of 2% lidocaine (0.15 ml/kg; Xylocaine; Astra, Astra Södertälje, Sweden) and ketamine (50 mg/ml; Ketalar; Parke-Davis, Morris Plains, NJ). All surgical procedures were performed using sterile techniques.

### Generation of choroidal neovascularization by laser photocoagulation

The CNV lesions were induced in rat eyes by laser photocoagulation as previously described [14,15]. Briefly, after the rats were anesthetized, pupils were dilated with 1% tropicamide (1% Mydriacyl; Alcon Laboratories, Hünenberg, Switzerland). A piece of cover glass served as a contact lens. Argon laser (Novus Omni; Coherent, Palo Alto, CA) irradiation was delivered through a slit lamp (Carl Zeiss, Oberkochen, Germany). Laser parameters were set as follows: spot size was 50 µm, power was 400 mW, and exposure duration was 0.05 s. An attempt was made to break Bruch’s membrane, as clinically evidenced by central bubble formation. Six lesions were created between the major retinal vessels in each fundus.

### Topical application of thioredoxin-vasostatin 48 eyedrops

Recombinant TRX-VS48 was purified and applied to eyes as previously described [21]. The TRX-VS48 and TRX eyedrops were formulated in PBS (1 μg/ml) and stored at 4 °C. Like methylcellulose solution, TRX-VS48 is stable in PBS without significant loss of integrity or anti-angiogenesic activity for at least 7 days. One day after CNV induction, the rats were treated topically with either TRX eyedrops or topical TRX-VS48 eyedrops (50 μl PBS each time, n=8) three times daily for 20 day.

### Quantification of choroidal neovascularization lesions by flat-mount analysis using fluorescein isothiocyanate-dextran labeling

The blood vessels in rat eyes were labeled by perfusion with fluorescein isothiocyanate (FITC)-dextran (2×10^6^ MW; Sigma) [7,22]. Briefly, approximately 50 ml of Lactated Ringer solution (Y F Chemical Corp., Taipei, Taiwan) was injected via the left ventricle, followed by 20 ml of FITC-dextran in lactated Ringer solution (5 mg/ml) in 10% gelatin. The eyes were removed and fixed for 2 h in 10% phosphate-buffered formalin. After the cornea and lens were removed, RPE-choroid-sclera flat mounts were obtained by hemisection of the eye and by peeling the neural retina away from the underlying RPE. The retina was detached and flat-mounted onto a slide. The fluorescence in flat mounts was examined by fluorescence microscopy, and images were digitized using a three-color charge-coupled device video camera and a frame grabber. The area of hyperfluorescence associated with each burn, the CNV lesion, was measured using Image-analysis software (Image J; National Institutes of Health, Bethesda, MD) by two independent ophthalmologists who were blind to the experimental design.

### Fundus fluorescent angiography

The CNV lesions were evaluated by fluorescent angiography (FA) analysis as previously described [14]. On days 21, 28, 35, and 42 after laser photocoagulation, the CNV lesions were evaluated by FA using a digital fundus camera (Visupac 450; Ziess FF450, Germany). The fluorescein sodium solution (10%; 0.1 ml/kg; Fluorescite; Alcon, Fort Worth, TX) was injected into the intraperitoneal cavity of the rats. Late-phase angiograms were obtained 8 min after injection, and digital fundus pictures of bilateral eyes were taken within 1 min. A choroidal neovascularization was defined as present when early hyperfluorescence with late leakage was present at the site of the laser injury. The area of CNV was measured using image analysis software (Retina Angiography Area Measurement program; Heidelberg Engineering, Germany) by two independent ophthalmologists who were blind to the experimental design.

### Histological analysis

After the final round of fluorescent angiography (FAG) analysis on day 42, the eyes were enucleated (n=8 rats per group), fixed for 30 min in 4% paraformaldehyde, embedded in OCT (Sakura, Tokyo, Japan), and sectioned to 10 μm thickness. The frozen sections were stained with hematoxylin and eosin for examination under light microscope (Olympus BX40) or immunofluorescence analysis von Willebrand factor (vWF) antibodies (anti-vWF; 1:50 dilution; Dako Denmark A/S, Glostrup, Denmark) followed by incubation with FITC-conjugated secondary antibodies. Immunofluorescence analysis was performed under fluorescent microscopy.

### Electroretinograms

The single bright flash electroretinograms (ERG; UTAS-E 300; LKC Technology, Gaithersburg, MD) under a dark-adapted environment were performed to assess either drug safety or retinal function changes following topical TRX-VS48 or TRX treatment after laser induced CNV. The ERG recordings were divided into two experimental groups. To assess TRX-VS48 safety, the first group of rats received topical TRX-VS48 three times per day in the right eye and topical PBS three times per day in the left eye for 3 weeks. The ERG for drug toxicity was performed on day 21. After the laser-induced CNV, the second group of rats received topical TRX-VS48 three times per day in the right eye and topical TRX three times per day in the left eye for 3 weeks. The ERG for comparison of retinal function between TRX-VS48 and TRX was performed on days 21, 28, 35, and 42. After at least a 1 h of darkness adaptation, rats were anesthetized. Gold foil was placed on the cornea with 2% methylcellulose gel (Omni Vision, Neuhausen, Switzerland). A reference electrode was attached to the shaven skin of the head and a ground electrode clipped to the rat’s ear. After reducing the background noise below 60 Hz, a single flash of bright light (duration, 100 ms), 30 cm from the eye, was used as the light stimulus. Responses were amplified with a gain setting ±500 μV and filtered with low 0.3 Hz and high 500 Hz from an amplifier. Data were acquired, digitized, and analyzed using EM for Windows, version 2.6 (UTAS-E 300; LKC Technology). The amplitude and latency of the a- and b-waves were measured and averaged.

### Statistical analysis

For cell analysis, differences between groups were analyzed by ANOVA with two-tailed probability. To analyze the differences between the different groups, a Mann–Whitney U test analysis with two-tailed probability was used, and a p value of less than 0.05 was considered significant. To analyze the differences between each eye of paired groups, a Wilcoxon signed-ranks test analysis with two-tailed probability was used, and a p value of less than 0.05 was considered significant. Results were representative of at least three independent experiments. Data were presented as mean±SD (standard deviation) or SEM (standard error of the mean). All statistical analyses were conducted using SPSS software, version 15.0.1 (SPSS Inc., Chicago, IL).

## Results

### Fused thioredoxin-vasostatin 48 inhibited the migration and tube formation of endothelial cells

**Figure 1:**
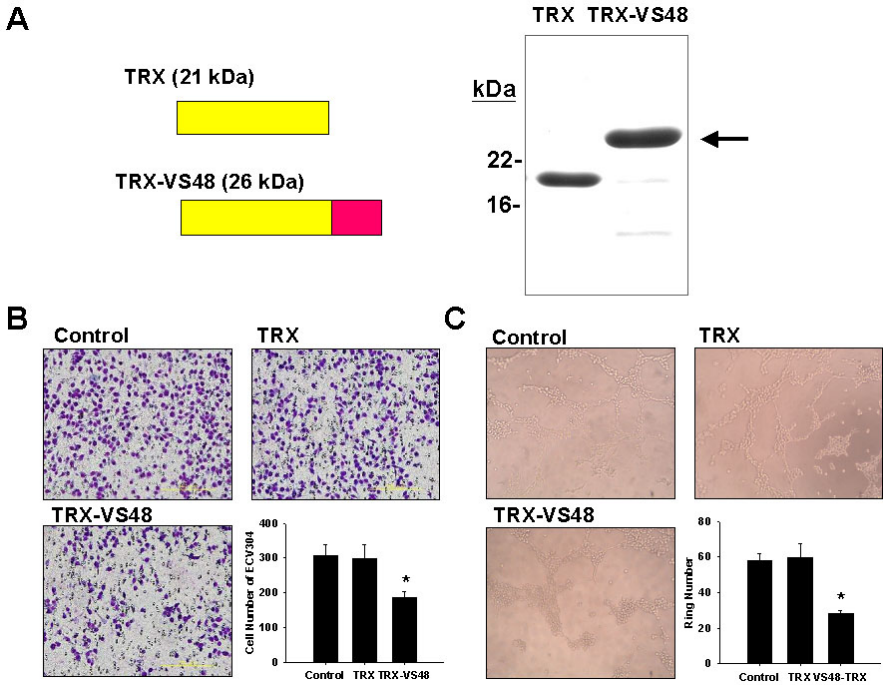
Effect of thioredoxinand thioredoxin-vasostatin 48 on angiogenic processes in cultured endothelial cells. **A**: Scheme represented thioredoxin (TRX) and TRX-fused VS48 (TRX-VS48). The purity of TRX and TRX-VS48 was analyzed by 10% sodium dodecyl sulfate polyacrylamide gel electrophoresis (SDS–PAGE) analysis. Recombinant TRX-VS48 is expressed and purified with a molecular weight of 26 kDa (arrow). **B**: Effect of TRX-VS48 on endothelial cell migration was assayed by the Boyden's chamber method. After treatment with TRX-VS48, the cell migration of ECV304 cells was significantly decreased. **C**: Tube formation of ECV304 cells was quantified on matrigel plates. After treatment with TRX-VS48, the tube formation of ECV304 cells was significantly decreased. Data represents the mean±SEM of quadruplicate experiments. *p<0.05.

Recombinant VS48 fused with thioredoxin (TRX-VS48) was expressed and purified (Figure 1A). Boyden's chamber studies found that TRX-VS48 inhibited the migration of ECV304 endothelial cells to 40%–50% of that in the control group (Figure 1B). Tube formation of ECV304 endothelial cells was evaluated by seeding on matrix gel plates, and it was found that TRX-VS48 inhibited the tube-forming capacity of ECV304 endothelial cells by 40%–50% of that of the control groups (Figure 1C).

### Fused thioredoxin-vasostatin 48 inhibited vessel growth in aorta rings

**Figure 2:**
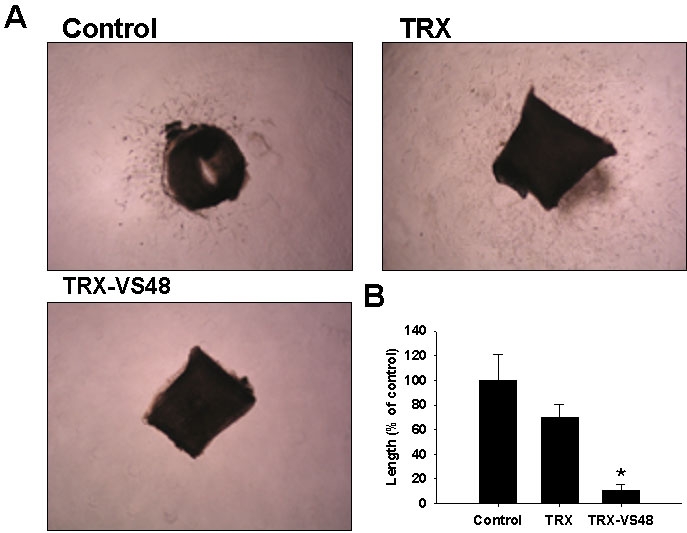
Effect of thioredoxin-vasostatin 48 on the vessel networks in rat aorta rings. **A**: The profile of micro-vessel outgrowth in rat aorta arteries treated with thioredoxin (TRX), TRX-fused vasostatin 48 (TRX-VS48), and control. After 5 days, the greatest distance from the aortic ring body to the end of the vascular sprouts was measured in three different rings per group. Magnification is 20×. **B**: Vessel sprouting was significantly attenuated by TRX-VS48. Data represents the mean±SD of quadruplicate experiments *(p<0.01).

The rat aortic ring assay was used to evaluate the angiogenic function of TRX-VS48 and TRX protein ex vivo. The aorta rings used were embedded in matrigel and cultured in MCDB 131 optimized medium for microvascular endothelial cells, to generate branching microvessels (Figure 2A). The sprouting of aorta vessels was significantly inhibited by TRX-VS48 (10.54±5.021%), compared with that of the control groups (100±20.65% for the PBS group and 70.01±10.32 for the TRX group, respectively; **p<0.01; Figure 2B).

### Alleviation of choroidal neovascularization through topical application of fused thioredoxin-vasostatin 48 by flat-mount analysis

**Figure 3:**
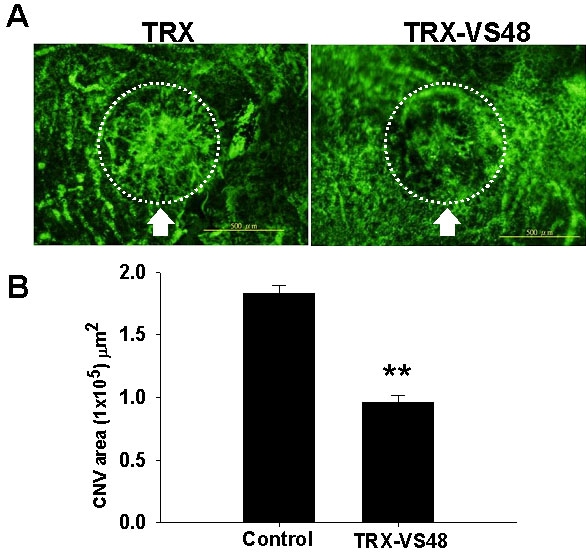
Flat-mount analysis of choroidal vascularity detected by fluorescein isothiocyanate-dextran staining in rat eyes after topical thioredoxin-vasostatin 48 application. The choroidal vascularity of laser-induced lesions in rat eyes was examined by injecting fluorescein isothiocyanate (FITC)-dextran. **A**: FITC-dextran positive blood vessels are presented in choroids of thioredoxin(TRX)-treated or TRX-fused vasostatin (TRX-VS48)-treated rats. Arrows indicate the laser-induced lesions. **B**: Quantification of FITC-dextran fluorescence in choroids of TRX-treated eyes or TRX-VS48-treated eyes are shown. Data are summarized as mean±SEM (n=8). Double asterisk (**) represents p<0.01.

To evaluate the therapeutic efficacy of TRX-VS48 eyedrops, the neovascularization in laser-induced CNV in rats after TRX-VS48 treatment was investigated using flat-mount analysis after perfusion with FITC-dextran on day 21 (n=10; Figure 3A). It was found that TRX-VS48 treatment significantly reduced the CNV area (0.096±0.005 mm^2^) compared with TRX (0.183±0.006 mm^2^; p<0.01; Figure 3B). Thus, topical TRX-VS48 application was effective in suppressing CNV.

### Diminished choroidal neovascularization through topical application of fused thioredoxin-vasostatin 48

**Figure 4:**
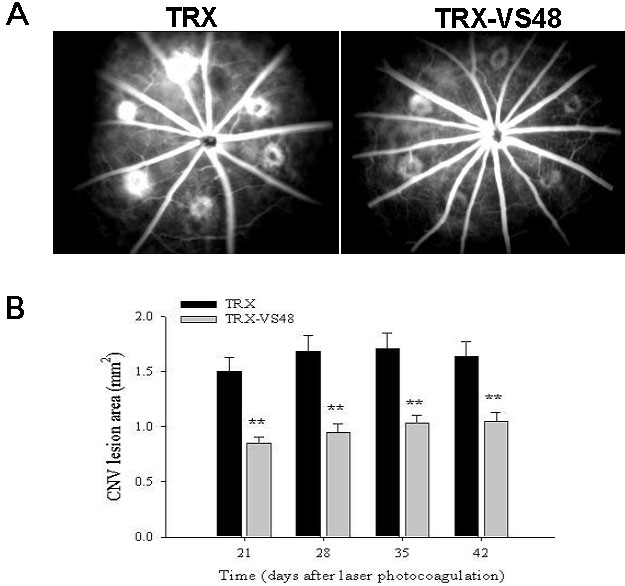
Suppression of laser photocoagulation-induced choroidal neovascularization by thioredoxin-vasostatin 48 eyedrops. **A**: Representative choroidal neovascularization (CNV) lesion in the eyes of rats treated with topical thioredoxin eyedrops (left) or topical thioredoxin-vasostatin 48 eyedrops (right) as identified by fluorescent angiography (FA) on day 21. **B**: The areas of CNV lesions were determined by FA examination on days 21, 28, 35, and 42 after laser photocoagulation. Data are summarized as mean + SEM (n=8). Double asterisk (**) represents p<0.01).

One day after CNV induction, the rats were treated with TRX or TRX-VS48 (n=10) on days 1 through 20. The CNV incidences in rats were evaluated by FA analysis on days 21, 28, 35, and 42 after CNV induction. TRX-VS48-treated eyes exhibited a significant reduction in CNV extents compared with TRX-treated eyes, as analyzed by FA (Figure 4). It was found that the TRX-VS48-treated eyes exhibited reduced the extent of CNV (Figure 4A). The CNV areas in TRX-VS48-treated rats were significantly decreased on day 21 (0.85±0.05 mm^2^ versus 1.50±0.12 mm^2^ for TRX-treated eyes, p<0.01), day 28 (0.95±0.07 mm^2^ versus 1.68±0.15 mm^2^ for TRX-treated eyes, p<0.01), day 35 (1.03±0.07 mm^2^ versus 1.71±0.14 mm^2^ for TRX-treated eyes, p<0.01), and day 42 (1.05±0.08 mm^2^ versus 1.63±0.14mm^2^ for TRX-treated eyes, p<0.01; Figure 4B). These results indicated that topical TRX-VS48 application attenuated the severity of experimental CNV despite a trend of declining efficacy.

### Alleviation of choroidal neovascularization and reduced subretinal neovascularization through topical vasostatin 48 application

**Figure 5:**
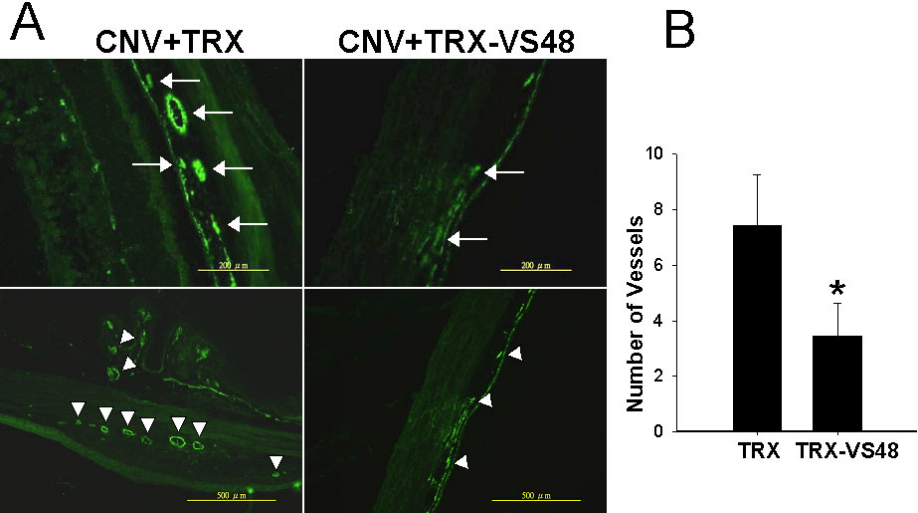
Immunofluorescence analysis of choroidal vascularity in rat eyes after topical thioredoxin-vasostatin 48 application. The choroidal vascularity in thioredoxin (TRX)-treated or TRX-fused vasostatin 48 (TRX-VS48)-treated eyes was examined by immunofluorescence analysis using anti-Von Willebrand factor (vWF). **A**: Representative profiles of vWF-positive vessels in retinochoroid of TRX-treated or TRX-VS48-treated eyes are shown. Arrows indicate big circles in high power field (larger arrows) and choroidal neovascularizationby little circles in low power field (arrowheads). **B**: Quantification pf vWF-positive blood vessels in choroids from TRX-VS48- or TRX-treated eyes. Data are mean±SEM (n=8) *(p<0.01).

To elucidate the therapeutic mechanism of TRX-VS48 eyedrops (1 μg/ml in PBS), we analyzed rat retinas by immunofluorescent methods. Examination of blood vessel density revealed that the number of vWF-positive blood vessels was significantly decreased in TRX-VS48 treated eyes, compared with TRX-treated ones (7.43±1.82 versus 3.43±1.21 per high power field; p<0.01; Figure 5). Therefore, topical VS48 application ameliorated laser-induced neovascularization, thereby disrupting CNV development.

### Retinal toxicity of topical application of fused thioredoxin-vasostatin 48

To evaluate the safety of TRX-VS48 eyedrops, the retinal function in control rats after topical TRX-VS48 treatment for 20 days was investigated using electroretinograms on day 21. There was no statistical difference in the latency and amplitude of the a-wave and b-wave between eyes with TRX-VS48 eyedrops and control eyes (n=8; Table 1). These results suggested that there was no obvious retinal toxicity after topical application of TRX-VS48.

**Table 1:** Electroretinogram values after topical application of VS48-TRX eye drops for 20 days.

**Electroretinogram parameters**	**VS48-TRX**	**PBS**	**p value**
a-wave amplitude, μV	204.1±20.5	239.5±30.4	0.474
a-wave latency, ms	20.4±0.9	21.1±2.4	0.778
b-wave amplitude μV	446.7±32.9	393.9±51.2	0.291
b-wave latency, ms	56.1±2.3	52.9±3.0	0.438

Retinal toxicity was evaluated by electroretinogram (ERG) recording. There was no statistical difference in the amplitude or latency of the ERG a-wave and b-wave between topical VS48-TRX and PBS treatment groups.

### Retinal function following topical application of fused thioredoxin-vasostatin 48 after laser-induced choroidal neovascularization

**Figure 6:**
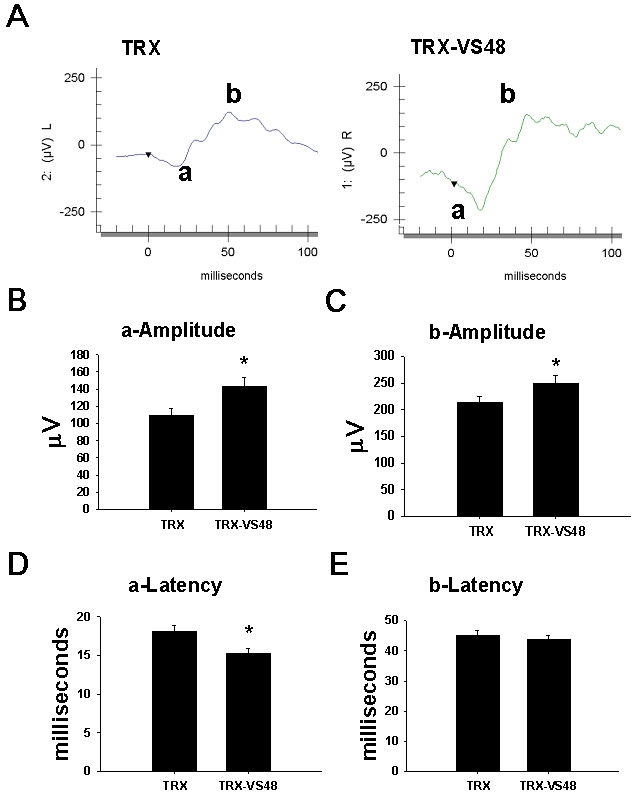
Electroretinogram analysis of the choroidal neovascularization-induced alterations in retina function after topical thioredoxin-vasostatin 48 application. The function of retina cells in choroidal neovascularization (CNV) rats was evaluated by electroretinogram (ERG) on day 35 after topical thioredoxin (TRX) and TRX-fused vasostatin 48 (TRX-VS48) treatment (n=24). **A**: The representative electroretinogram tracings of TRX-treated (left panel) and TRX-VS48-treated (right panel) eyes. **B**: Effect of TRX-VS48 on a-wave amplitude. The a-wave amplitude significantly increased in the TRX-VS48 eyes *(p<0.05). **C**: Effect of TRX-VS48 on b-wave amplitude. The b-wave amplitude significantly increased in the TRX-VS48 eyes; *(p<0.05). **D**: Effect of TRX-VS48 on a-wave latency. The a-wave latency was significantly decreased in the TRX-VS48 eyes *(p<0.05). **E**: Effect of TRX-VS48 on b-wave latency. No significant difference in the b-wave latency was found between TRX and TRX-VS48 groups.

To evaluate the therapeutic effects of TRX-VS48 eyedrops, the functional integrity of the retina in rats after laser photocoagulation following TRX-VS48 or TRX treatment for 20 days was also investigated using electroretinograms (n=24). The CNV eyes showed significantly decreased a-wave amplitude, prolonged a-wave latency, and decreased b-wave amplitude on day 35. The ERG results of TRX-VS48-treated and TRX-treated eyes on day 35 are shown in Figure 6. Topical TRX-VS48 reversed the CNV-induced alterations in retina function by ERG on day 35 (Figure 6A). ERG revealed that the amplitudes of the a-wave and b-wave were significantly increased in TRX-VS48 eyes (p<0.05; Figure 6B,D). ERG revealed that the latency of the a-wave was significantly decreased in TRX-VS48 eyes (p<0.05; Figure 6C).

## Discussion

Rodent models of laser induced CNV are now extensively used to identify angiogenic proteins and to evaluate the efficacy of antigangiogenic therapies [5,7,14,23-25]. This is true for gene-based therapies, because the ultimate goal is to achieve a long-lasting alteration of gene expression [2,24]. The present study provided proof-of-principle evidence that topical TRX-VS48 application suppresses laser induced choroidal neovascularization in rats. Vasostatin (VS), the N-terminal domain (amino acids 1–180) of calreticulin, is a potent angiogenesis inhibitor isolated from culture supernatants of an Epstein-Barr virus-immortalized cell line. Recombinant VS was shown to inhibit bFGF- and VEGF-induced endothelial cell proliferation in vitro [17]. VS primarily inhibits endothelial cell proliferation in tumor models by inducing endothelial apoptosis and arresting the cell cycle at the G1 phase [17]. In addition, several potential mechanisms are proposed for this antiangiogenic activity, including the generation of free radicals such as superanions and nitric oxide [26] and upregulation of the Fas/FASL system. In our previous studies, intramuscular VS gene delivery led to elevated VS levels in the blood, which profoundly inhibited tumor growth and CNV in animals [14,18]. It was found that VS is stable in an eyedrop solution at 4 °C for at least 7 days without losing activity [21].

There are several advantages of VS48 over other endogenous angiogenesis inhibitors. First, VS48 is a smaller peptide fragment than VS, and it is highly soluble and stable in solution, which makes it suitable for eyedrop formulation and storage. Second, the anti-angiogenic potency of VS48 is similar to that of VS, which is higher than that of endostatin or angiostatin [17,27]. Third, like VS, VS48 exhibits minimal cytotoxicity to eye cells, including the retina and iris pigment epithelial cells [14,19]. Finally, topical VS application does not induce inflammation or have other adverse effects in animals [15]. In fact, a recent report indicated that VS is anti-inflammatory, which might contribute to CNV suppression [28]. Electroretinogram analysis also showed that topical TRX-VS48 application did not alter retinal functions, suggesting that topical TRX-VS48 is likely safe for the treatment of age-related macular degeneration. Furthermore, topical TRX-VS48 improved the amplitude of the a- and b-waves and the latency of the a-wave, compared with topical TRX on day 35 after laser photocoagulation.

Topical application is an advantageous route for drug delivery to the retina because it is non-invasive and results in minimal adverse effects from its systemic administration or intraocular injection. Repeated long-term intravitreal delivery runs the risk of significant complications such as retinal detachment, endophthalmitis, vitreous hemorrhage, and cataract formation [16]. Some drugs have even been shown to successfully reach the vitreous cavity for treatment of retino-choroidal diseases following topical application [29,30]. We demonstrated the inhibition of CNV through the topical application of angiogenesis inhibitor VS in a previous study [15]. In this study, we have demonstrated that TRX-VS48 suppressed the tube formation and migration of endothelial cells. At present, the explanation remains elusive about how the topically applied TRX-VS48 protein exerts its anti-angiogenic functions upon the choroid inside the eyes. One possibility is that VS48 penetrates or diffuses through the subconjunctiva or sclera, and then reaches the choroid. For penetration, eyedrop components have to conquer the barrier of the cornea, as well as other blood-ocular barriers. Therefore, efficacy may depend on the lipid solubility and size of the active molecule. Future studies are required to delineate the tissue distribution, therapeutic mechanism, and half-life of TRX-VS48 in vivo. Because it takes years or even decades for CNV development in humans, it is debatable whether the protein or its gene vector should be employed to achieve long-term prevention or suppression of CNV. We have investigated the feasibility of intramuscular, polymer-based VS gene therapy for long-term CNV suppression [14]. However, systemic VS expression may bring potential risks to the cardiovascular system or to physiologic processes such as wound healing. Due to the usage of a constitutive promoter, another drawback for gene therapy is the inability to stop the production of the gene product when side effects occur. Despite the short half-life of the protein in vivo, topical TRX-VS48 administration has the following advantages over gene therapy: (1) safety via a local route, (2) feasibilityof extending or terminating the therapeutic program, and (3) flexibility in modifying the therapeutic dose [15]. In the present study, the efficacy of the TRX-VS48 eyedrops indeed diminished as the experiments progressed, probably due to the lack of continued supply of the therapeutic protein.

In conclusion, the present study revealed the therapeutic potential of topical TRX-VS48 administration to attenuate the development of CNV in rat eyes. The data from non-treated CNV eyes were not included because the CNV lesions in TRX-treated eyes were similar to those in non-treated CNV eyes. Since none of the experimental animals showed overt adverse effects, TRX-VS48 eyedrops may constitute a novel alternative for CNV treatment. Thus, TRX-VS48 may serve as an adjuvant therapy in conjunction with current VEGF-targeting and therapeutic agents such as Avastin or Ranibizumab for long-term CNV management. Future studies on the optimal dosages, therapeutic mechanisms, and pharmacological kinetics of VS48 are warranted to further evaluate the potential to translate these pre-clinical results into clinical applications for CNV treatment.
